# Analysis of Hospital Quality Measures and Web-Based Chargemasters, 2019: Cross-sectional Study

**DOI:** 10.2196/26887

**Published:** 2021-08-19

**Authors:** Kunal N Patel, Olena Mazurenko, Eric Ford

**Affiliations:** 1 Northern Illinois University DeKalb, IL United States; 2 Indiana University Indianapolis, IN United States; 3 University of Alabama at Birmingham Birmingham, AL United States

**Keywords:** chargemaster, standard charge, price transparency, health care, diagnosis-related group, DRG, quality measures, the Centers for Medicare and Medicaid Services regulation, CMS

## Abstract

**Background:**

The federal health care price transparency regulation from 2019 is aimed at bending the health care cost curve by increasing the availability of hospital pricing information for the public.

**Objective:**

This study aims to examine the associations between publicly reported diagnosis-related group chargemaster prices on the internet and quality measures, process indicators, and patient-reported experience measures.

**Methods:**

In this cross-sectional study, we collected and analyzed a random 5.02% (212/4221) stratified sample of US hospital prices in 2019 using descriptive statistics and multivariate analysis.

**Results:**

We found extreme price variation in *shoppable* services and significantly greater price variation for medical versus surgical services (*P*=.006). In addition, we found that quality indicators were positively associated with standard charges, such as mortality (β=.929; *P*<.001) and readmissions (β=.514; *P*<.001). Other quality indicators, such as the effectiveness of care (β=−.919; *P*<.001), efficient use of medical imaging (β=−.458; *P*=.001), and patient recommendation scores (β=−.414; *P*<.001), were negatively associated with standard charges.

**Conclusions:**

We found that hospital chargemasters display wide variations in prices for medical services and procedures and match variations in quality measures. Further work is required to investigate 100% of US hospital prices posted publicly on the internet and their relationship with quality measures.

## Introduction

### Background

Increases in health care expenditures have persisted throughout the years in the United States despite policy efforts to *bend the curve*. According to the Centers for Medicare and Medicaid Services (CMS), the US health care spending in 2018 increased 4.6% from the previous year and totaled US $3.6 trillion [1]. A contributing factor to the rise in health care expenditures comes from the fact that hospitals do not compete on price in the same way other efficient product markets do (such as the e-commerce sector). Currently, there are differences in what is charged by health care systems compared with what is paid by consumers [2]. Subsequently, consumers are, in effect, *price takers* accepting the hospital charges negotiated with their insurer [3].

As a result, historically, consumers have not been as price-sensitive toward making health care decisions when compared with consumer decision-making behaviors commonly observed in other economic sectors. With the continual increase in US health care spending, a widely held view is that greater consumer engagement in health care will help hold prices down. In turn, greater consumer engagement will slow down the sector’s expansion rate if (and when) consumers place a substantial emphasis on making price-sensitive decisions using pricing transparency information [4,5]. To that end, the CMS have issued 2 regulations that require hospitals to increase their *price transparency* [6,7]. The first regulation required hospitals to disclose their diagnosis-related group (DRG) chargemasters on the web publicly in a machine-readable form (such as a *Microsoft Excel* file) starting in 2019. Releasing the DRG chargemasters on the internet was met with little resistance from hospitals, as the information did not compromise revealing negotiated hospital pricing strategies vis-à-vis third-party payors or competitors. Although there was little resistance to the first federal regulation, previous literature has shown an abundance of nonprice-transparent and noncompliant hospitals and hospitals with inaccessible pricing information [8-10].

Nonetheless, understanding newly available US chargemaster information is vital to patients because American patients are sent a medical bill after receiving treatment. A medical bill will contain the patient’s portion owed of hospital standard charges for medical services and procedures that were delivered net of any contractual allowances and third-party payments. Therefore, standard charges are relevant to the consumer, either directly by influencing their purchase decisions before receiving medical care or indirectly when they receive a medical bill after treatment.

### Objective

This study aims to assess the variability of publicly available DRG chargemaster data and its relation to quality measures, process indicators, and patient experience measures as a source of information for consumer quality assessment and price-sensitive decision-making purposes. The research benefits three audiences. For policy makers, this study provides an early assessment of the pricing transparency regulation’s utility. For researchers, being able to collect and compare hospitals’ pricing data is an important task if they are to inform policy maker efforts on controlling health care spending. In addition, researchers can inform the public at large and assist other stakeholders, such as nongovernmental organizations, in providing an analysis of pricing information found on chargemasters that is understandable. Finally, for health care administrators, this research can shed new light on the importance of presenting standard charges to the public in compliance with the law.

## Methods

### Procedures

We conducted a cross-sectional study of web-based publicly available hospital chargemasters from 2019. First, we assessed the descriptive statistics and coefficients of variation (CVs) to describe the standard charges grouped by the DRG code. We aimed to describe the full extent of price variability in hospital standard charges.

We then performed 2 median chi-square tests on standard charges and type of service (either medical or surgical). Median chi-square tests were performed because the standard charges were not normally distributed, that is, standard charges were skewed to the right. The first test was for average standard charge (either above the median or below the median) by the type of service (either medical or surgical). Similarly, the second test was for the CV (either above the median or below the median) by the type of service (either medical or surgical).

Next, we performed a log-linear, ordinary least squares regression model to fit the natural log-transformed standard charges on hospital characteristics. We log-transformed the dependent variable (standard charges) owing to the right-skewness and lack of normal distribution. We removed outliers with residuals of IQR 1.5 below the first quartile or IQR 1.5 above the third quartile. β coefficients, *P* values, and robust SEs were presented as predictors. Robust SEs were clustered on hospital to correct for related observations. All analyses were conducted using *Microsoft Excel* and *Stata/SE 15.1*. The institutional review board of the University of Alabama at Birmingham exempted this study.

### Data Source

We retrieved chargemasters from hospital websites on the internet between August 25, 2019, and October 3, 2019, if they were formatted using DRG primary codes (eg, chargemasters in Healthcare Common Procedure Coding System or common procedural terminology primary code were excluded). In line with previous research, we obtained common hospital characteristic data from the Hospital Compare, CMS, American Hospital Association (AHA), and Hospital Consumer Assessment of Healthcare Providers and Systems (HCAHPS).

### Sampling Strategy

**Figure 1:**
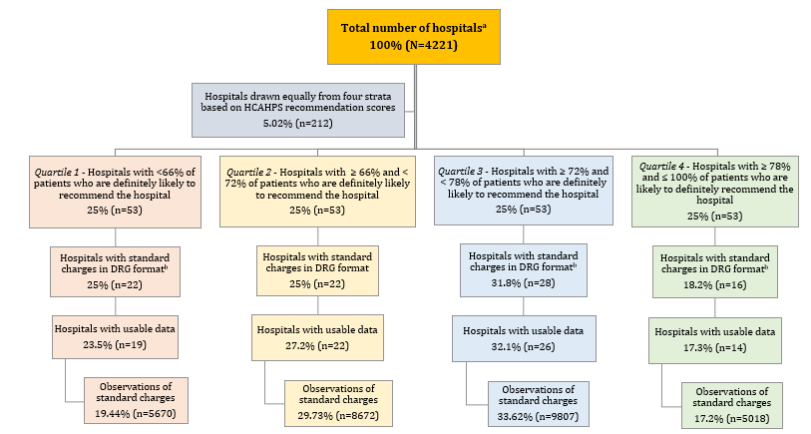
Data sampling strategy. ^a^Total number of hospitals drawn from Hospital Consumer Assessment of Healthcare Providers and Systems (HCAHPS) survey from 3rd quarter of 2018. ^b^Some hospitals provided data in an unusable format, such as in the All Patient Refined–diagnosis-related group coding format vs Medicare Severity–diagnosis-related group, providing maximum or minimum charges vs standard charges, etc. DRG: diagnosis-related group.

We constructed a random, stratified sample to assess US hospitals (Figure 1). It has been previously shown that hospital website quality is associated with HCAHPS recommendation scores [11]. Thus, to ensure an adequate variation of low- to high-quality websites, we stratified hospitals (n=4221) listed in HCAHPS data from October 1, 2017, to September 20, 2018, into 4 ranked quartiles based on the measure, “Patients who reported YES, they would definitely recommend the hospital.” In total, 1.26% (53/4221) of hospitals were randomly selected from each of the 4 strata, representing a total of 5.02% (212/4221) of the hospitals in the HCAHPS data set. The sample size was restricted to maintain the feasibility of manual data collection and processing costs [12]. In sum, we had 29,167 observations of standard charges grouped by 81 different hospitals.

### Predictors for Hospital Characteristics

Quality predictors included benchmark measures for the hospital’s overall rating (hospital rating categories: 1 star, 2 stars, 3 stars, 4 stars, 5 stars, and missing), mortality rate, safety score, readmission rate, effectiveness of care score, efficient use of medical imaging score, and patient experience score. These measures and their categorical values (either below the national average, same as the national average, above the national average, or missing) were obtained using the Hospital General Information data set from the CMS. In addition, we included one additional patient experience measure: the likelihood of patients to recommend the hospital using the quartile categories described in the *Sampling Strategy* section (1=lowest quartile and 2, 3, and 4=highest quartile).

Controls included hospital ownership type (government—hospital district or authority, government—local, physician, proprietary, voluntary nonprofit—church, voluntary nonprofit—other, and voluntary nonprofit—private). Next, using data from the AHA Annual Survey, the hospital bed size (6-24 beds, 25-49 beds, 50-99 beds, 100-199 beds, 200-299 beds, 300-399 beds, 400-499 beds, and 500 or more beds) was controlled. Previous work has used the number of competitors in the market as a measure of competition (rather than the Herfindahl-Hirschman index) [13]. In line with these studies, we calculated a control measure for competition using the number of Medicare providers per 5-digit ZIP code (1=least and 2 and 3=most) from the HCAHPS data set. The DRG primary code was controlled using individual dummies for each of the DRG primary codes from the CMS data set for Medicare Severity–DRG version 36. Finally, 2 geographical control variables were included using the AHA Annual Survey data set. They were regions (New England, Mid Atlantic, South Atlantic, East North Central, East South Central, West North Central, West South Central, Mountain, and Pacific as defined by the AHA Annual Survey) and US states (individual dummies for each US state).

## Results

### Variance Analysis

CMS specifically defined 5 services using DRG primary codes to be *shoppable* in a forthcoming regulation on increasing health care price transparency (effective January 1, 2021). We present the price variability for these 5 *shoppable* medical services for our 5.02% (212/4221) sample of US hospitals in Table 1. The shoppable medical services included in our analysis were sorted from most to least variable, as measured by the CV. The maximum standard charge was frequently many orders of magnitude higher than the minimum. For example, the standard charge for DRG primary code 473 *cervical spinal fusion without comorbid conditions or major comorbid conditions or complications* had a mean (SD) value of US $89,302 (SD US $50,122), a CV of 0.561, and ranged from US $30,924 to US $249,283. The maximum standard charge for the procedure was over US $210,000 more than the minimum standard charge out of 44 hospitals that performed the service.

**Table 1:** Standard charges for Centers for Medicare and Medicaid Services–specified shoppable services^a^.

Medicine and surgery services^b^	DRG^c^ primary code	Hospitals observed (n=212), n (%)	Price variability (US $)		Coefficient of variation (SD divided by mean)
			Mean (SD)	Minimum-maximum, range	
Major joint replacement or reattachment of lower extremity without MCC^d^	470	75 (35.4)	68,329 (41,724)	26,401-224,052	0.611
Spinal fusion except cervical without MCC	460	50 (23.6)	123,744 (71,755)	30,995-427,374	0.580
Cervical spinal fusion without CC^e^ or MCC	473	44 (20.8)	89,302 (50,122)	30,924-249,283	0.561
Cardiac valve and other major cardiothoracic procedures with cardiac catherization with MCC	216	28 (13.2)	430,274 (195,719)	139,460-912,194	0.455
Uterine and adnexa procedures for nonmalignancy without CC or MCC	743	62 (29.2)	41,338 (18,662)	11,863-87,981	0.451

^a^The table is sorted from most to least variable using the coefficient of variation.

^b^These are the only 5 selected services using the diagnosis-related group primary code (as opposed to the common procedural terminology or Healthcare Common Procedure Coding System) that the Centers for Medicare and Medicaid Services determined to include in the forthcoming regulation (effective date January 1, 2021), which mandates public disclosure of payer-specific negotiated charges, deidentified minimum and maximum negotiated charges, and discounted cash prices for at least 300 shoppable services, including 70 Centers for Medicare and Medicaid Services–specified shoppable services and 230 hospital-selected shoppable services.

^c^DRG: diagnosis-related group.

^d^MCC: major comorbid conditions or complications.

^e^CC: comorbid conditions.

Next, out of the set of 761 DRG primary codes, the data for the most and least variable services by type of service (either medical or surgical) with at least 30 observations are presented in Table 2. Most and least variable services were measured by the highest and lowest CVs, respectively. Noticeably, it appears that surgical procedures had higher means and lower CVs, which is investigated further in the *Standard Charge and Type of Service* section.

**Table 2:** Top 10 most and least variable services for diagnosis-related group primary codes^a^.

Medicine and surgery services (rank)		DRG^b^ primary code	Type	Hospitals (n=212), n (%)	Price variability (US $)				Coefficient of variation (SD divided by mean)		Magnitude of range (US $)
					Mean (SD)	Minimum-maximum, range					
**Top 10 most variable services**											
	Normal newborn (1)	795	Medical	57 (26.9)	27,052 (167,415)	1005	1,268,646	6.189		1,267,641	
	Reticuloendothelial and immunity disorders with MCC^c^ (2)	814	Medical	30 (14.2)	129,016 (376,201)	13,470	2,077,708	2.916		2,064,238	
	Skin ulcers with CC^d^ (3)	593	Medical	43 (20.3)	44,730 (80,382)	6668	493,015	1.797		486,346	
	Other infectious and parasitic diseases diagnoses with MCC (4)	867	Medical	32 (15.1)	96,332 (170,037)	8608	962,984	1.765		954,377	
	Other respiratory system operating room procedures without CC or MCC (5)	168	Surgical	38 (17.9)	75,296 (109,621)	16,330	695,556	1.456		679,226	
	Neonates, died or transferred to another acute care facility (6)	789	Medical	50 (23.6)	91,278 (129,536)	1839	687,641	1.419		685,802	
	Other endocrine, nutritional, and metabolic operating room procedures with MCC (7)	628	Surgical	32 (15.1)	159,266 (209,529)	41,388	1,188,069	1.316		1,146,681	
	Other factors influencing health status (8)	951	Medical	52 (24.5)	19,934 (22,603)	32	109,262	1.134		109,230	
	Minor skin disorders without MCC (8)	607	Medical	57 (26.9)	28,153 (30,979)	5013	226,300	1.100		221,287	
	Depressive neuroses (10)	881	Medical	44 (20.8)	21,152 (21,902)	3144	140,972	1.035		137,829	
**Top 10 least variable services**											
	Percutaneous intracardiac procedures without MCC (1)	274	Surgical	36 (17)	112,404 (43,667)	46,738	255,453	0.388		208,715	
	Kidney and ureter procedures for neoplasm with CC (2)	657	Surgical	38 (17.9)	78,905 (31,783)	23,587	156,374	0.403		132,787	
	Other heart assist system implant (3)	215	Surgical	30 (14.2)	335,647 (139,272)	153,355	702,998	0.415		549,643	
	Ischemic stroke, precerebral occlusion, or transient ischemia with thrombolytic agent with CC (4)	62	Medical	39 (18.4)	84,117 (34,993)	27,457	187,137	0.416		159,680	
	Cardiac pacemaker revision except device replacement with CC (5)	261	Surgical	31 (14.6)	68,581 (30,005)	23,154	130,492	0.438		107,338	
	Vaginal delivery with sterilization and/or dilation and curettage (6)	767^e^	Surgical	50 (23.6)	24,912 (10,938)	8210	54,446	0.439		46,236	
	Cesarean section without CC or MCC (7)	766^e^	Surgical	56 (26.4)	24,106 (10,687)	9737	58,931	0.443		49,194	
	Disorders of the biliary tract without CC or MCC (8)	446	Medical	59 (27.8)	28,628 (12,805)	7936	72,899	0.447		64,963	
	Uterine and adnexa procedures for nonmalignancy without CC or MCC (9)	743	Surgical	62 (29.2)	41,338 (18,662)	11,863	87,981	0.451		76,118	
	Aortic and heart assist procedures except pulsation balloon with MCC (10)	268	Surgical	31 (14.6)	251,216 (113,721)	92,804	623,820	0.453		531,016	

^a^Only services in the diagnosis-related group primary code with at least 30 observations are included. Most and least variable services are measured by the highest and lowest coefficients of variation, respectively.

^b^DRG: diagnosis-related group.

^c^MCC major comorbid conditions or complications.

^d^CC: comorbid conditions.

^e^Diagnosis-related group (DRG) codes 766 and 767 have been removed from Medicare Severity–DRG version 36.

### Standard Charge and Type of Service

The relationship between standard charge and type of service (medical or surgical) was assessed for significant differences using 2 different median chi-square tests. The tables are presented in the top and bottom panels of Table 3. In both median chi-square tests, the cells represent the counts of individual DRG primary codes. The median chi-square test for average standard charge versus the type of service was significant (Pearson *χ*^2^_1_ [sample size=758]=284.1; *P*<.001). The observed number of average standard charges was significantly greater than the expected number for surgical services, with average standard charges greater than the median (observed=309 and expected=193). The median chi-square test for CV by type of service was also significant (Pearson *χ* ^2^_1_ [sample size=758]=7.6; *P*=.006). However, in contrast to average standard charges, the observed number of CVs was significantly less than the expected number of CVs for surgical services, with CVs greater than the median. In summary, surgical services (as opposed to medical services) generally tended to have significantly more average standard charges and fewer CVs above the median.

**Table 3:** Contingency table for average standard charge versus the type of service and coefficient of variation for standard charge versus the type of service^a^.

Standard Charge		Type of service								Total (n=758), n (%)
		Medical				Surgical				
		Observed (n=372), n (%)	Expected (n=372), n (%)	Chi-square contribution	Observed (n=386), n (%)		Expected (n=386), n (%)	Chi-square contribution		
**Average**										
	Less than median	302 (79.7)	186 (50)	72.3	77 (20.3)		193 (50)	69.7	379 (50)	
	Greater than median	70 (18.5)	186 (50)	72.3	309 (81.5)		193 (50)	69.7	379 (50)	
**Coefficient of variation (SD divided by mean)**										
	Less than median	167 (44.1)	186 (50)	1.9	212 (55.9)		193 (50)	1.9	379 (50)	
	Greater than median	205 (54.1)	186 (50)	1.9	174 (45.9)		193 (50)	1.9	379 (50)	

^a^Counts are individual diagnosis-related group primary codes, for example, 70 medical-type diagnosis-related group codes have averages greater than the median standard charge.

### Hospital Characteristics of Standard Charges

We examined standard charges across hospital characteristics: hospital ownership, hospital rating, mortality, safety, readmission, effectiveness of care, patient experience, competition, efficient use of medical imaging, patient recommendation, region, bed size, US state, and DRG primary code (Table 4). Using multivariate regression modeling after removing outliers, we found that our model was able to explain nearly 90% of the variation in the randomized, stratified sample of standard charges in 2019 using categorical variables for the predictors (Table 5).

**Table 4:** Hospital characteristics of included standard charges (N=29,167)^a^.

Variables				Hospital, n (%)
**Hospital ownership**				
	**Government**			
		Hospital district or authority	1534 (5.26)	
		Local	1197 (4.1)	
	Physician			150 (0.51)
	Proprietary			9021 (30.93)
	**Voluntary nonprofit**			
		Church	1390 (4.77)	
		Other	4102 (14.06)	
		Private	11,773 (40.36)	
**Hospital rating**				
	1 star (worst)			2336 (8.01)
	2 stars			9363 (32.1)
	3 stars			6003 (20.58)
	4 stars			9459 (32.43)
	5 stars (best)			1903 (6.52)
	Missing			103 (0.35)
**Mortality**				
	Below the national average			4474 (15.34)
	Same as the national average			17,696 (60.67)
	Above the national average			5926 (20.32)
	Missing			1071 (3.67)
**Safety**				
	Below the national average			8619 (29.55)
	Same as the national average			5695 (19.53)
	Above the national average			13,633 (46.74)
	Missing			1220 (4.18)
**Readmission**				
	Below the national average			15,237 (52.24)
	Same as the national average			2975 (10.20)
	Above the national average			10,388 (35.62)
	Missing			567 (1.94)
**Effectiveness of care**				
	Below the national average			4383 (15.03)
	Same as the national average			23,394 (80.21)
	Above the national average			1287 (4.41)
	Missing			103 (0.35)
**Patient experience**				
	Below the national average			11,932 (40.91)
	Same as the national average			8296 (28.44)
	Above the national average			8537 (29.27)
	Missing			402 (1.38)
**Competition**				
	1 (least)			22,625 (77.57)
	2			4360 (14.95)
	3 (most)			2182 (7.48)
**Efficient use of medical imaging**				
	Below the national average			4936 (16.92)
	Same as the national average			16,343 (56.03)
	Above the national average			6028 (20.67)
	Missing			1860 (6.38)
**Patient recommendation**				
	1 (lowest quartile)			5670 (19.44)
	2			8672 (29.73)
	3			9807 (33.62)
	4 (highest quartile)			5018 (17.2)
**Region**				
	New England			1384 (4.75)
	Mid Atlantic			2845 (9.75)
	South Atlantic			4616 (15.83)
	East North Central			4359 (14.94)
	East South Central			2713 (9.3)
	West North Central			1083 (3.71)
	West South Central			4797 (16.45)
	Mountain			2766 (9.48)
	Pacific			4604 (15.78)
**Number of beds**				
	6-24			329 (1.13)
	25-49			1017 (3.49)
	50-99			3049 (10.45)
	100-199			6017 (20.63)
	200-299			4650 (15.94)
	300-399			5249 (17.99)
	400-499			4057 (13.91)
	500 or more			4799 (16.45)

^a^The table does not show values for each category for US state and diagnosis-related group primary code.

**Table 5:** Regression results for standard charges in a sample of US hospitals (N=27,530)^a^.

Variables				β (robust SE)		*P* value
**Hospital ownership**						
	**Government**					
		Hospital district or authority	.856 (0.104)		<.001	
		Local	.499 (0.159)		.002	
	Physician			−1.879 (0.257)		<.001
	Proprietary			.828 (0.177)		<.001
	**Voluntary nonprofit**					
		Church	1.008 (0.089)		<.001	
		Other	Reference		N/A^b^	
		Private	.172 (0.111)		.13	
**Hospital rating**						
	1 star (worst)			.499 (0.212)		.02
	2 star			.422 (0.076)		<.001
	3 star			Reference		N/A
	4 star			.133 (0.185)		.47
	5 star (best)			.109 (0.207)		.60
	Missing			−.042 (0.756)		.96
**Mortality**						
	Below the national average			.514 (0.051)		<.001
	Same as the national average			Reference		N/A
	Above the national average			.244 (0.125)		.05
	Missing			.961 (0.188)		<.001
**Safety**						
	Below the national average			−.085 (0.046)		.06
	Same as the national average			Reference		N/A
	Above the national average			.102 (0.063)		.11
	Missing			−.007 (0.187)		.97
**Readmission**						
	Below the national average			.929 (0.158)		<.001
	Same as the national average			Reference		N/A
	Above the national average			.578 (0.147)		<.001
	Missing			−1.023 (0.325)		.002
**Patient experience**						
	Below the national average			−.046 (0.055)		.41
	Same as the national average			Reference		N/A
	Above the national average			.294 (0.075)		<.001
	Missing			3.707 (0.346)		<.001
**Effectiveness of care**						
	Below the national average			.081 (0.102)		.43
	Same as the national average			Reference		N/A
	Above the national average			−.919 (0.119)		<.001
**Efficient use of medical imaging**						
	Below the national average			−.277 (0.055)		<.001
	Same as the national average			Reference		N/A
	Above the national average			−.458 (0.128)		.001
	Missing			.321 (0.084)		<.001
**Patient recommendation**						
	1 (lowest quartile)			Reference		N/A
	2			−.236 (0.066)		.001
	3			−.169 (0.074)		.03
	4 (highest quartile)			−.414 (0.072)		<.001
**Competition**						
	1 (least)			Reference		N/A
	2			.546 (0.065)		<.001
	3 (most)			.552 (0.119)		<.001
**Number of beds**						
	6-24			Reference		N/A
	25-49			2.063 (0.287)		<.001
	50-99			1.518 (0.261)		<.001
	100-199			1.832 (0.32)		<.001
	200-299			2.63 (0.341)		<.001
	300-399			2.082 (0.286)		<.001
	400-499			2.013 (0.294)		<.001
	500 or more			2.075 (0.262)		<.001
Constant				10.004 (0.418)		<.001

^a^The table shows the results for a log-linear regression using the natural log function to transform the dependent variable, that is, standard charge. Other covariates for individual state code and diagnosis-related group Code Dummy Variables are not shown. Outliers with residuals IQR 1.5 below the first quartile or IQR 1.5 above the third quartile are omitted. Robust SEs are clustered on hospital to correct for related observations. Standard charges are in dollars. R^2^=0.8955 and number of observations=27,530.

^b^N/A: not applicable.

All quality indicators were associated with standard charges at the statistically significant α=.05 level, except for the patient safety indicator. The 2 quality indicators associated with the largest significant *increases* in standard charges were below the national average mortality rate (β=.929; *P*<.001) and below the national average readmission rate (β=.514; *P*<.001); they were associated with 153% and 67% significantly higher standard charges on average, respectively, compared with the national average groups, holding other factors constant. On the contrary, the three quality indicators associated with the largest significant *decreases* in standard charges in our study were above the national average effectiveness of care (β=−.919; *P*<.001), above the national average efficient use of medical imaging (β=−.458; *P*=.001), and the highest quartile patient recommendation scores (β=−.414; *P*<.001); they were associated with 60%, 37%, and 34% significantly lower standard charges on average, respectively, than those of the reference groups, holding other factors constant.

Finally, for Table 5, please note that the interpretations of β coefficients were on average, while holding all else constant and using natural log-transformed standard charges as the outcome variable. In addition, the constant and β coefficients for the missing categories in the relevant variables were not described but can be found in the table. Robust SEs were clustered on hospital to correct for related observations. Outliers were removed as described in the *Methods* section, leaving 27,530 observations in the regression model. Overall, a large amount of variation in standard charges was explained by our regression model (R^2^=89.55%).

## Discussion

### Principal Findings

Wide differences exist between hospital billed charges and the amount of money that hospitals expect to receive for services [14]. Our analysis found that chargemaster DRG prices on the internet varied greatly between facilities. At a minimum, the web-based chargemaster data do not reflect the marginal cost of performing 1 instance of a procedure. Different hospitals have widely varying fixed costs that may drive the variance to some extent, but this is not sufficient to explain the differences observed [15]. A more plausible explanation is that there are systematic differences in the business strategies related to chargemaster construction, as found in our analysis.

Reviewing Table 2, even the procedures with *relatively* low SDs and CVs had wide enough ranges to indicate that there is little to no relation to the chargemaster’s rates and actual underlying costs. For example, a previous study on Ohio state data from 2007 to 2012 showed that a hospital with the highest median charge for a normal newborn delivery (DRG primary code: 795) could be nearly 4 times as costly as the hospital with the lowest median charge despite no differences in length of stay (which typically is 2 days) [16]. We found even further drastic differences in our data set of the standard charge of a normal newborn delivery on a national level when compared with this study, where the maximum standard charge for the procedure was more than 1250 times greater than the minimum standard charge. Furthermore, our finding for the *Normal Delivery of a Newborn* service having the largest variation among our data was unusual for three reasons. First, the mean standard charge was relatively small, which usually leads to lower variances. Next, the upper bound of US $1,268,646 defied any reasonable expectations for this service. Finally, the minimum rate, US $1005, also defied logic. Even an uncomplicated delivery typically involves a 2-day stay with a per diem above US $1400, which would total more than US $2800 for the charge [13]. Additional examples of standard charges with wide ranges for the exact same service are commonly found in the literature [17-19].

Thereafter, we sought to test the differences in variability in the type of service (either medical or surgical). The estimated CVs for surgical-type DRGs were significantly smaller than those for medical DRGs using standard charge data for Maryland between 1979 and 1981 [20]. However, as the DRG patient diagnosis classifications are refined overtime, variation among medical-type DRGs could potentially converge toward the more favorable lower levels of variation of surgical-type DRGs [20]. However, we found that after 4 decades of revisions to DRG codes, where the number of unique codes increased from ≥400 in the 1980s to ≥700 in the 2020s, medical-type DRGs still had more variability than surgical-type DRGs. Our results may indicate challenges, as the results show that it is still increasingly more difficult to predict medical-type standard charges that have more variability when compared with surgical services. As a result, health care providers and other stakeholders will have to work increasingly harder to assist consumers in making informed decisions, especially for medical services.

Afterward, we sought to understand whether the wide variances observed were systematically related to hospital characteristics for quality performance indicators. A number of hospital characteristics were shown to be significantly associated with standard charges, including physical characteristics such as bed size or ownership structure, geographical characteristics, controls for the service or procedure code, competition, and quality indicators (such as patient recommendation scores or readmission rates).

Overall, our results were largely consistent with those of a previous study that found that standard charges in hospital chargemasters were well predicted using hospital characteristics [21]. However, the previous study did not find sufficient evidence that hospitals with higher prices also provided a higher quality of care [21]. In contrast to this finding, we found 2 key quality characteristics to be positively and significantly associated with standard charges (when controlling for market competition, physical characteristics, geographical differences, and DRG primary code in the multivariate analysis): mortality rates and readmission rates. Furthermore, these quality indicators are consistent with economic theory, where higher quality goods and services demand a higher price in the competitive market [22].

On the other hand, there is not a singular positive or negative relationship between price and quality, and at times, price and quality can either have a positive or negative relationship [23]. We found 3 health care quality indicators with contradictory results to standard economic theory: effectiveness of care, efficient use of medical imaging, and patient recommendation scores. In other words, as quality increases, the standard charge decreases, which is a contradictory pricing behavior.

Complexities exist in modern health care, which causes gaps in the ability of health care systems to deliver consistent, effective, and efficient care [24]. Therefore, significant undertreatment and overtreatment occur [24]. A possible explanation for the relationship between higher quality effectiveness of care and efficient use of medical imaging being associated with decreases in standard charges is that they lower waste, and thus, they reduce standard charges. Finally, a potential reason for higher patient recommendation scores being associated with lower standard charges is that the hospital may benefit from increased volume (or demand owing to more patient referrals) and, in turn, from economies of scale.

At this juncture, it is important to digress from the first phase of health price transparency regulation and discuss the implications of the second phase briefly to shed some light on other implications of this study in the context of present health care systems and policies. Although hospitals provide chargemaster data, the *standard charges* rarely provide information for patients to make informed health care decisions [25]. As a large number of patients are insured, they are more interested in cost sharing information and specific insurer-negotiated pricing rather than standard charges for health care services. Therefore, the second round of CMS transparency regulations more broadly requires hospitals to disclose the rates they have negotiated with third-party payers for service bundles starting in 2021 [7], including the following:

- Gross charge: the charge for an individual item or service that is reflected on a hospital’s chargemaster, absent any discounts
- Discounted cash price: the charge that applies to an individual who pays cash or cash equivalent for a hospital item or service
- Payer-specific negotiated charge: the charge that a hospital has negotiated with a third-party payer for an item or service
- Deidentified minimum negotiated charges: the lowest charge that a hospital has negotiated with all third-party payers for an item or service
- Deidentified maximum negotiated charges: the highest charge that a hospital has negotiated with all third-party payers for an item or service.

Patients may use this additional information in 2021 to more accurately price-shop, insurers may use this information to bargain for better reimbursement rates, and other facilities may use this information to alter their pricing strategies and compete more effectively in the more transparent health care market. Therefore, this information is closely guarded by health plans [26]. Thus, hospitals, insurers, lobbying groups, and other stakeholders oppose this regulation because the negotiated prices have immeasurable proprietary strategic value, and disclosure thereof will have far-reaching implications on both price and quality competition. It remains to be seen if health systems will be able to block or alter the second phase of price transparency regulations before the scheduled implementation in 2021.

### Limitations

While conducting this study on health care price transparency, there are 2 important limitations that need to be discussed. First, we did not analyze pricing information from other coding systems, such as common procedural terminology, Healthcare Common Procedure Coding System, or other proprietary formats. Some hospitals published chargemasters using other codes that were not mandated. Thus, the study results can only be generalized to the extent that DRG codes bundle services together correctly and correspond accurately to services rendered for patients. Some of these other coding systems rely on billing specialists to itemize services rendered, and they may or may not result in more accurate pricing, which could be higher or lower on average when compared with DRG-coded charges we analyzed in this study. However, the DRG coding system is one of the most widely used systems for preparing patient bills, and the results of this study are directly applicable to this most commonly used hospital pricing system in the United States.

Second, we did not follow up, investigate, or verify individual observations of standard charges. It is possible (and quite likely) that hospital chargemasters unintentionally contain outdated, erroneous, or inaccurate standard charges. These mistakes may have been published on the web for the public unbeknownst to hospital administrators. We mitigated these effects as much as possible by using statistical techniques where appropriate, such as analyzing median values and removing outliers.

### Conclusions

Patients are not solely influenced by costs when making health care decisions; they base their decisions on several factors, including the opinions and information supplied by their health care providers and insurers. Moreover, previous literature has shown that patients just do not want to be a cog in the health care system, but in reality, they want to share in the decision-making processes regarding where to seek treatment with their health care providers [27,28]. Such health care–related decisions are commonly determined based on the quality of available medicals goods and services at a particular facility or by a specific provider. Therefore, patient decisions to seek treatment are being determined jointly by providers and consumers using both clinical quality and out-of-pocket cost information.

In summary, the results of this cross-sectional study, which analyzed the pricing behavior at hospitals in the first phase of the price transparency regulations, draw attention to the fact that policy makers, researchers, and health care administrators as well as, ultimately, consumers all need to be vigilant about health care price transparency and its relation to quality measures. There was extreme variation in *shoppable* services. Findings unearthed in this study include: one of the most commonly performed services (normal newborn delivery) had the most variation, significantly larger variation existed in medical services than surgical services, and quality variables were associated either positively or negatively with standard charges. It is ever more important for all the parties involved, such as researchers, policy makers, and health care administrators, to act in good faith and make the information as user-friendly and accessible as possible as well as use this information to the highest, fullest potential—bending the health care cost curve.

### Future Directions

It is crucial for researchers, policy makers, and health care administrators to work together to design a holistic registry or database system to document these chargemasters. This study has demonstrated the potential value of such information using publicly available chargemaster data on the internet from a cross-sectional random, stratified sample of 5.02% of the US hospitals. This process can be scaled up to collect, clean, and document chargemasters for all US hospitals multiple times per year, such as quarterly or semiannually.

## Abbreviations

AHA: American Hospital Association
CMS: Centers for Medicare and Medicaid Services
CV: coefficient of variation
DRG: diagnosis-related group
HCAHPS: Hospital Consumer Assessment of Healthcare Providers and Systems

## References

[1] Historical. Centers for Medicare & Medicaid Services (CMS).

[2] Babcock P (2019). Paralyzed by prices: an analysis of price theory within the context of health care. Linacre Q.

[3] Malakhov S (2017). Moral hazard, optimal healthcare-seeking behavior, and competitive equilibrium. Expert J Econ.

[4] Kullgren JT, Duey KA, Werner RM (2013). A census of state health care price transparency websites. J Am Med Assoc.

[5] Gusmano MK, Maschke KJ, Solomon MZ (2019). Patient-centered care, yes; patients as consumers, no. Health Aff (Millwood).

[6] FY 2019 LTCH PPS final rule. Centers for Medicare & Medicaid Services (CMS).

[7] (2019). CY 2020 Hospital Outpatient Prospective Payment System (OPPS) policy changes: hospital price transparency requirements. Centers for Medicare & Medicaid Services (CMS).

[8] Mullens CL, Hernandez JA, Anderson ED, Allen L (2020). Just because (most) hospitals are publishing charges does not mean prices are more transparent. JMIR Med Inform.

[9] Lu A, Chen E, Vutam E, Brandt J, Sadda P (2020). Price transparency implementation: accessibility of hospital chargemasters and variation in hospital pricing after CMS mandate. Healthc (Amst).

[10] Patel K, Rucks A, Ford E, Hefner JL, Al-Amin M, Huerta TR, Aldrich AM, Griesenbrock TE (2020). Consumers' exposure to price transparency: compliance testing and sentiment analysis of US hospitals during 2019. Transforming Health Care (Advances in Health Care Management, Vol. 19).

[11] Ford EW, Huerta TR, Diana ML, Kazley AS, Menachemi N (2013). Patient satisfaction scores and their relationship to hospital website quality measures. Health Mark Q.

[12] Zuckerman S, Hadley J, Iezzoni L (1994). Measuring hospital efficiency with frontier cost functions. J Health Econ.

[13] Ghiasi A, Zengul FD, Ozaydin B, Oner N, Breland BK (2017). The impact of hospital competition on strategies and outcomes of hospitals: a systematic review of the U.S. Hospitals 1996-2016. J Healthc Fin.

[14] Muhlestein D (2013). What types of hospitals have high charge-to-reimbursement ratios?. Health Affairs Blog.

[15] Boncheck L (2019). Will more transparency help us lower the cost of health care?. J Lancaster Gen Hosp.

[16] Hall ES, Lang MD, Levin L, Narendran V (2015). Variation in neonatal inpatient charges at the state and local level. Hosp Top.

[17] Agarwal A, Dayal A, Kircher SM, Chen RC, Royce TJ (2020). Analysis of price transparency via National Cancer Institute-designated cancer centers' chargemasters for prostate cancer radiation therapy. JAMA Oncol.

[18] Sprehe MR, Johnson R (2016). Using chargemaster data to understand childhood leukemia costs. Blood.

[19] White C, Whaley C (2019). Prices paid to hospitals by private health plans are high relative to medicare and vary widely: findings from an employer-led transparency initiative. RAND Corporation.

[20] Frank RG, Lave JR (1985). The psychiatric DRGs. Are they different?. Med Care.

[21] Batty M, Ippolito B (2017). Mystery of the chargemaster: examining the role of hospital list prices in what patients actually pay. Health Aff (Millwood).

[22] Robinson JC (1988). Hospital quality competition and the economics of imperfect information. Milbank Q.

[23] Fitzgerald MP, Yencha C (2018). A test of policy makers’ formal and lay theories regarding health care prices. J Public Pol Mark.

[24] Oakes AH, Patel MS (2020). A nudge towards increased experimentation to more rapidly improve healthcare. BMJ Qual Saf.

[25] Glover M, Whorms D, Singh R, Almeida RR, Prabhakar AM, Saini S, Rosenkrantz AB (2020). A radiology-focused analysis of transparency and usability of top U.S. hospitals' chargemasters. Acad Radiol.

[26] Koller CF, Khullar D (2019). The commercial differential for hospital prices: responses from states and employers. J Am Med Assoc.

[27] Coulter A, Entwistle V, Gilbert D (1999). Sharing decisions with patients: is the information good enough?. Br Med J.

[28] Fowler Jr FJ, Levin CA, Sepucha KR (2011). Informing and involving patients to improve the quality of medical decisions. Health Aff (Millwood).

